# Visual-auditory integration for visual search: a behavioral study in barn owls

**DOI:** 10.3389/fnint.2015.00011

**Published:** 2015-02-13

**Authors:** Yael Hazan, Yonatan Kra, Inna Yarin, Hermann Wagner, Yoram Gutfreund

**Affiliations:** ^1^Department of Neuroscience, The Ruth and Bruce Rappaport Faculty of Medicine and Research Institute, TechnionHaifa, Israel; ^2^Department of Zoology and Animal Physiology, Institute for Biology II, RWTH Aachen UniversityAachen, Germany

**Keywords:** saliency, saccades, multisensory, visual search, barn owls, selective attention, sound localization

## Abstract

Barn owls are nocturnal predators that rely on both vision and hearing for survival. The optic tectum of barn owls, a midbrain structure involved in selective attention, has been used as a model for studying visual-auditory integration at the neuronal level. However, behavioral data on visual-auditory integration in barn owls are lacking. The goal of this study was to examine if the integration of visual and auditory signals contributes to the process of guiding attention toward salient stimuli. We attached miniature wireless video cameras on barn owls’ heads (OwlCam) to track their target of gaze. We first provide evidence that the area centralis (a retinal area with a maximal density of photoreceptors) is used as a functional fovea in barn owls. Thus, by mapping the projection of the area centralis on the OwlCam’s video frame, it is possible to extract the target of gaze. For the experiment, owls were positioned on a high perch and four food items were scattered in a large arena on the floor. In addition, a hidden loudspeaker was positioned in the arena. The positions of the food items and speaker were changed every session. Video sequences from the OwlCam were saved for offline analysis while the owls spontaneously scanned the room and the food items with abrupt gaze shifts (head saccades). From time to time during the experiment, a brief sound was emitted from the speaker. The fixation points immediately following the sounds were extracted and the distances between the gaze position and the nearest items and loudspeaker were measured. The head saccades were rarely toward the location of the sound source but to salient visual features in the room, such as the door knob or the food items. However, among the food items, the one closest to the loudspeaker had the highest probability of attracting a gaze shift. This result supports the notion that auditory signals are integrated with visual information for the selection of the next visual search target.

## INTRODUCTION

An animal in its environment is constantly bombarded by sensory input, while the animal can only orient and react to one object or event at a time. Therefore, mechanisms have evolved to select the most behaviorally relevant stimulus at any particular time. This brain process is called saliency mapping (Itti and Koch, 2000) and it lies at the base of selective attention. Animals tend to respond and attend to the stimulus which they perceive as the most salient (Luck and Ford, 1998).

It is widely accepted that animals compute a dynamic saliency value to different locations in space based on a combination of external factors, such as stimulus intensity, stimulus history, spatial context, etc., and internal factors, such as cognitive biases, behavioral tasks, reward history, motivations, etc. (Fecteau and Munoz, 2006). Therefore, the saliency of a stimulus is not a physical feature but rather a perceived feature dependent strongly on the context, history, surroundings, and internal state of the animal (Dutta and Gutfreund, 2014). It has been shown that the relationship between visual and auditory signals is an important external factor determining the saliency of stimuli in cats, primates, and humans (Stein et al., 1988; Driver and Spence, 1998; Recanzone, 2009). Congruent visual and auditory stimuli (same location and same time) are more likely to attract the animal’s gaze and attention, i.e., more salient, compared to unimodal stimuli or incongruent stimuli (Stein and Meredith, 1993; Frassinetti et al., 2002; Stein and Stanford, 2008). This process of combining visual and auditory signals is called visual-auditory integration. Thus, an animal is said to integrate visual and auditory information if the response to a combined stimulus is different from the response to each stimulus alone (Stein et al., 2014).

At the single neuron level, neurons that respond to both visual and auditory signals (bimodal neurons) have been identified in numerous levels of the nervous system (Beauchamp, 2005; Sugihara et al., 2006; Wallace et al., 2006; Kayser et al., 2007). Among these, the superior colliculus (SC), a mid-brain structure believed to be involved in selective attention, has been the most studied (Wallace et al., 1996). In cats and primates, many of the neurons in the SC have been shown to integrate auditory and visual signals in ways that mirror the behavioral observations, i.e., neurons respond maximally to visual and auditory signals that are congruent in time and space (Meredith and Stein, 1986; Meredith et al., 1987). These findings support the notion that the SC circuitry combines visual and auditory signals to perform saliency mapping in bimodal environments (Boehnke and Munoz, 2008; Mysore and Knudsen, 2011; Dutta and Gutfreund, 2014).

In non-mammalian species, physiological studies of visual-auditory integration for selective attention have been carried out mostly in the optic tectum (OT), the homolog of the SC. Among these, the OT of the barn owl consists of numerous bimodal neurons, integrating signals to enhance responses to congruent bimodal events, particularly if such events are surprising (Zahar et al., 2009). It has been suggested that this type of response facilitates the detection of salient stimuli (Reches et al., 2010; Gutfreund, 2012). The OT of barn owls possesses the most robust and accurate map of auditory space in any animal species studied so far (Knudsen, 1987). This auditory map is aligned with a precise retinotopic visual map (Knudsen, 1982). In addition, mechanisms of stimulus selection in the OT have been studied extensively in barn owls (Reches and Gutfreund, 2008; Mysore et al., 2010; Mysore and Knudsen, 2013). Therefore, this species has a great potential of being used as a research model for the study of visual-auditory integration for saliency mapping. Despite previous studies in barn owls on this subject at the neuronal level (Gutfreund et al., 2002; Reches and Gutfreund, 2009; Zahar et al., 2009; Reches et al., 2010), behavioral characterization of visual-auditory integration at the behavioral level is scarce (Whitchurch and Takahashi, 2006). The goal of this study is to contribute to such characterization and to develop new ways of studying visual-auditory integration at the behavioral level.

The perceived saliency of objects in the environment is manifested, in many species, in their visual search behavior (Wolfe and Horowitz, 2004; Hayhoe and Ballard, 2005; Berman and Colby, 2009). Visual search is the process of actively scanning the environment. Many animal species possess a small retinal area with a higher density of photoreceptors known as area centralis (in some species, this area is accompanied by an anatomical dipping in the retina, in which case it is called fovea). Such animals tend to shift their gaze so that points of interest will be acquired by this specialized retinal area. Animals exhibiting such a behavior are called foveating animals. It is widely accepted that the target at the retinal center in foveating animals is correlated with the focus of attention (Eckstein, 2011). Thus, by tracking the scan path of an animal in its environment, it is possible to obtain information on what objects and conditions are likely to attract the animal’s attention, i.e., are perceived as salient. This experimental procedure of gaze tracking has been used widely to study attention in humans and other species (Reinagel and Zador, 1999; Harmening et al., 2011; MacInnes et al., 2014). Gaze tracking can be technically difficult particularly when performed in freely moving animals, since it requires the exact measurement of both eye and head orientation as well as its relationship with the structure of the environment.

Barn owls possess a tubular eye structure that limits eye movement. Thus, in contrast to most other foveating animals, barn owls do not move their eyes in the orbits, maintaining a mostly fixed eye position relative to the head (Steinbach and Money, 1973; du Lac and Knudsen, 1990). Instead, they compensate for this lack by prominent head motions (Masino and Knudsen, 1990; Ohayon et al., 2006). This makes them an attractive animal model for the study of attention and visual search because it is not necessary to measure eye relative to head movements. It has been demonstrated unequivocally that barn owls are foveating targets, i.e., they use a single retinal location to acquire targets of interest (Ohayon et al., 2008; Harmening et al., 2011). The retina of barn owls contains a single area centralis but no visible fovea (Wathey and Pettigrew, 1989). It remains an open question whether the functional fovea in barn owls corresponds with the anatomically defined area centralis. In this study, we took advantage of the lack of eye movement and the spontaneous visual search behavior of barn owls. We attached miniature video cameras to the heads of barn owls in order to track the scan path and points of interests in the environment. In the first part, we show that the functional fovea corresponds with the area centralis. In the second part, we show that sounds influence visual search behavior in ways that support visual-auditory integration for saliency mapping.

## MATERIALS AND METHODS

### ANIMALS

Three adult barn owls (*Tyto alba*) were used in multiple test sessions. The owls were hatched in the breeding colony in the Technion’s Rappaport Faculty of Medicine and were hand-raised by lab members. Before the experiments, the owls were accustomed to the experimental room by being maintained in the room, on a perch, for about 2 h a day for a period of about 2 weeks. To increase the owls’ motivation to search spontaneously during the experiments, they were only fed on the perch at the end of each experiment from the food items on the floor. However, body weight was maintained normal. All procedures were approved by the Technion’s Committee for the Ethical Use of Laboratory Animals.

### VIDEO CAMERA ATTACHMENT AND RECORDINGS

**FIGURE 1:**
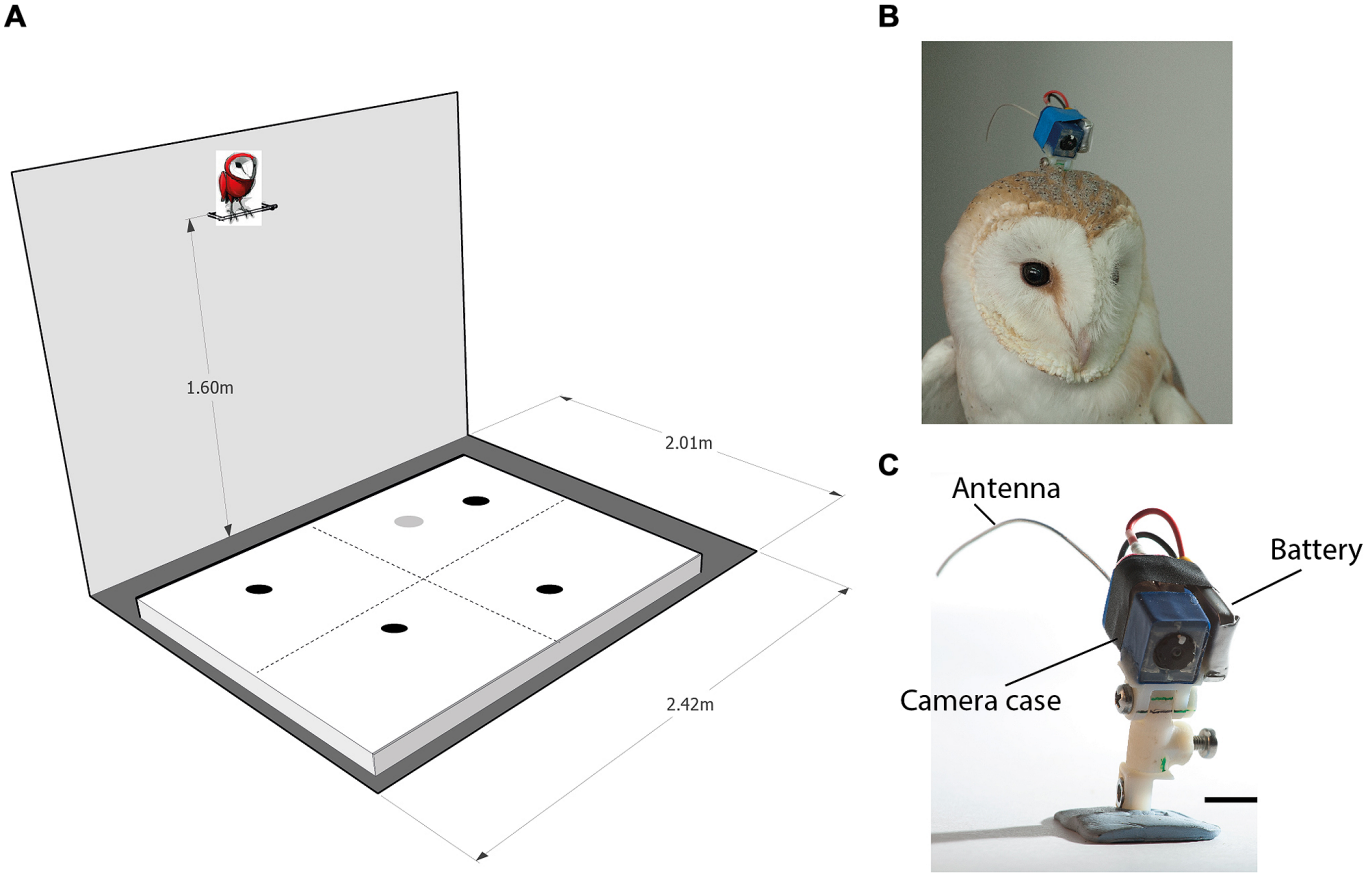
**The experimental apparatus. (A)** A scheme of the experimental room. An arena 200 cm × 160 cm is shown positioned on the floor. The perch, positioned 160 cm above the ground adjacent to the long wall, is shown with a cartoon owl on top of it. The dashed lines on the arena surface designate imaginary lines that divide the arena to four equal quadrants. The dark spots on the arena designate possible positions of four food items. Items were spread so that each quadrant will contain one item. The gray spot on the arena designates a possible location of the loud speaker. **(B)** An owl with the OwlCam attached to its head. **(C)** A close view of the OwlCam with the attachment unit and the battery in place. The scale bar designates 10 mm.

The OwlCam used was similar to the OwlCam developed and used by Harmening et al. (2011). It is devised from a miniature microcamera combined with a video broadcasting chip (900 MHz) and a rechargeable lithium-polymer battery [for more details on the camera, see Harmening et al. (2011)]. The OwlCam was attached to the head using a 3D printed attachment unit designed in the lab. One part of the unit was attached permanently to the skull bone with dental cement in a single surgical procedure. The other part, glued to the camera, was screwed to the permanent part at the beginning of each experiment and removed at the end of the experiment. The attachment unit was designed to allow the adjustment of the OwlCam orientation with respect to the head and to maintain a locked rigid positioning of the camera. The total weight of the device including the battery and attachment unit was 10.5 g and the dimensions of the camera case was 10 mm × 10 mm × 20 mm (**Figures 1B,C**). Owls wearing the mount showed no apparent behavioral changes including normal posture and flying.

The OwlCam delivered a wireless black and white video signal at 30 frames per second with an effective vertical resolution of about 380 lines and a view angle of about 60°. The video signal was collected with an off-the-shelf tunable video receiver (positioned about 2 m from the owl) and digitized at a resolution of 480 × 720 pixels for offline analysis.

### EXPERIMENTAL SET-UP

The experiments were performed in a 200 cm × 240 cm room. Although the room was stripped of any furniture, it was a regular lab room with some salient features on its walls such as electrical outlets, a dark door and a window covered with black paper. Moderate illumination was provided by a ceiling-mounted bulb. A 30 cm long perch was mounted to the wall opposite the door at a height of 160 and 10 cm away from the wall (see **Figure 1A** for a sketch of the experimental room). During the experiments, the owls were attached to the perch with a leg leash that was long enough to allow free walking and turning on the perch but prevented the owls from flying off the perch. A 160 cm × 200 cm wooden frame covered with a white cloth (the arena) was positioned on the floor centered under the perch. Before the beginning of the experiment, a curtain was drawn to block the owl’s view of the floor. Then, several food items (dead black lab mice on petri dishes) were scattered on the arena. At this point, the experimenter would leave the room and the curtain was drawn from outside the room with a string to reveal the arena to the owl.

**FIGURE 2:**
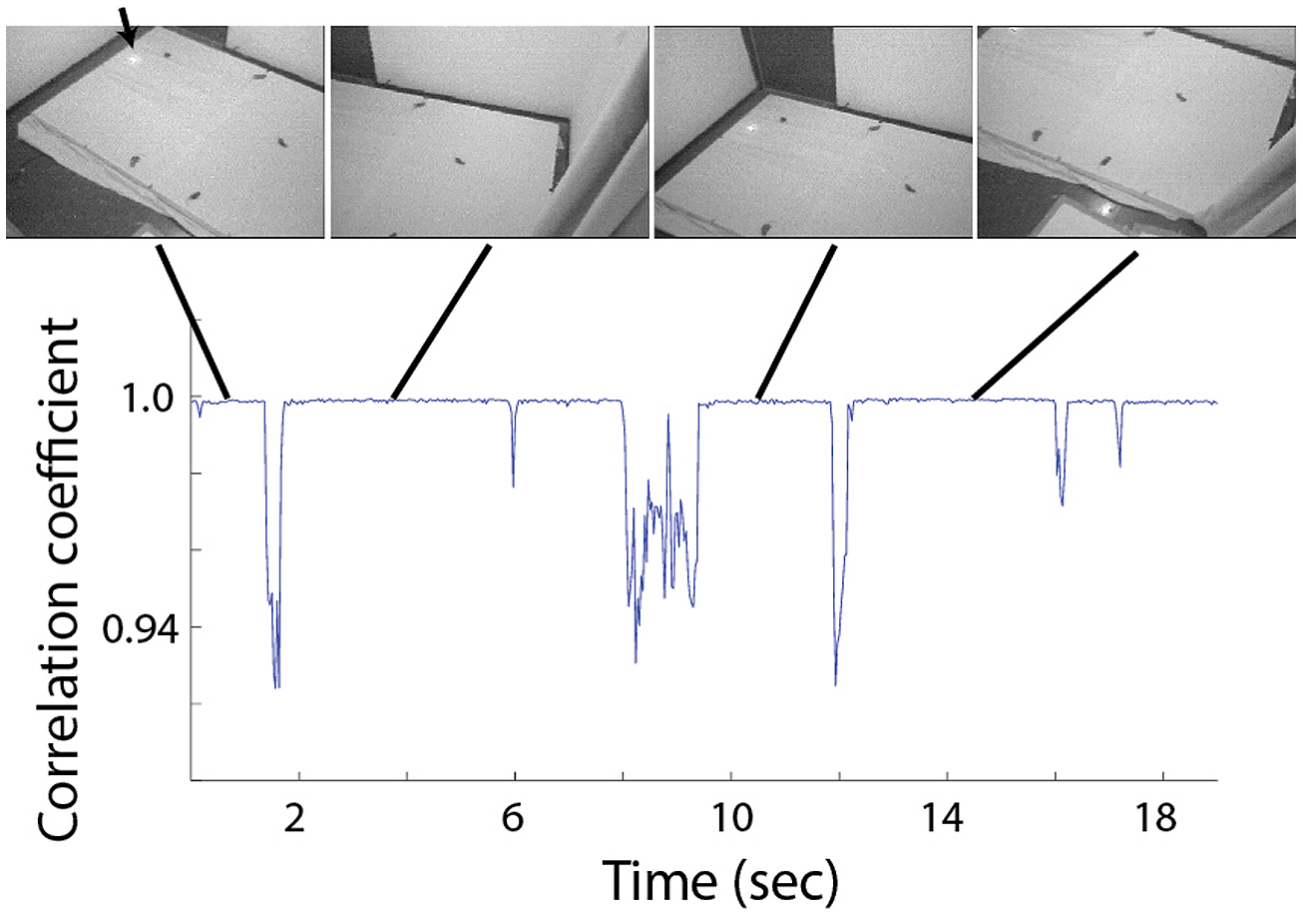
**Correlation between adjacent frames during 19 s of typical visual search behavior.** Four video frames extracted from periods of fixation are shown above. Each shows a different view of the experimental arena and the scattered food items. The arrow points to the position of the loudspeaker, visible via the infra-red light source attached to it.

In several of the experiments, a loudspeaker was positioned under the cloth, hidden from the owls. To register the position of the loudspeaker in the video frames, we attached an infrared LED to the speaker. The light emitted by the LED was clearly visible in the video (see arrow in **Figure 2**) but invisible to humans and barn owls (Netser et al., 2010). The loudspeaker was used to generate short and unexpected auditory stimuli. The auditory stimuli were stored on a PC connected to a Tucker-Davies Technologies (TDT) system III (~100 kHz sampling rate; 24 bit A/D), running custom Matlab programs. In order to reduce habituation to the sound, we used a library of 18 playbacks of natural sounds such as rustling leaves, animal sounds, etc. The different sounds varied in amplitude, frequency, temporal structure and duration (300–800 ms). Sounds were generated manually by a button pressed about once every 2 min. Each button press generated one randomly chosen sound from the library. The volume of all sounds was adjusted so that all were clearly audible to human listeners in the room. To synchronize the video recordings with the times of the auditory stimulation, an electronic switch was triggered from the sound-generating system to temporarily switch off the power supply to the OwlCam receiver. This resulted in about 5–6 disrupted frames in the video sequence, signaling the onset of the auditory stimulation. The disrupted frames were detected offline.

### DATA ANALYSIS

The behavior of the owls was mostly characterized by abrupt head movements and prolonged fixation period where the image was stable (see **Figure 2** and Ohayon et al., 2008). From each fixation period a single frame was extracted for further analysis. Fixation periods were identified manually by viewing the video sequence frame by frame and identifying stable periods in which the point of view doesn’t change. For visualization of the behavior (**Figures 2** and **6**) we used a frame by frame correlation function. Each frame in the video sequence was first passed through an edge detection filter, creating a reduced black and white image, the frames where then divided into an array of 8 × 10 rectangulars; the average value of all pixels in each rectangular was measured, creating 80 pixels images, each of which was correlated with its preceding frame and the correlation coefficient as a function of time was obtained.

**FIGURE 3:**
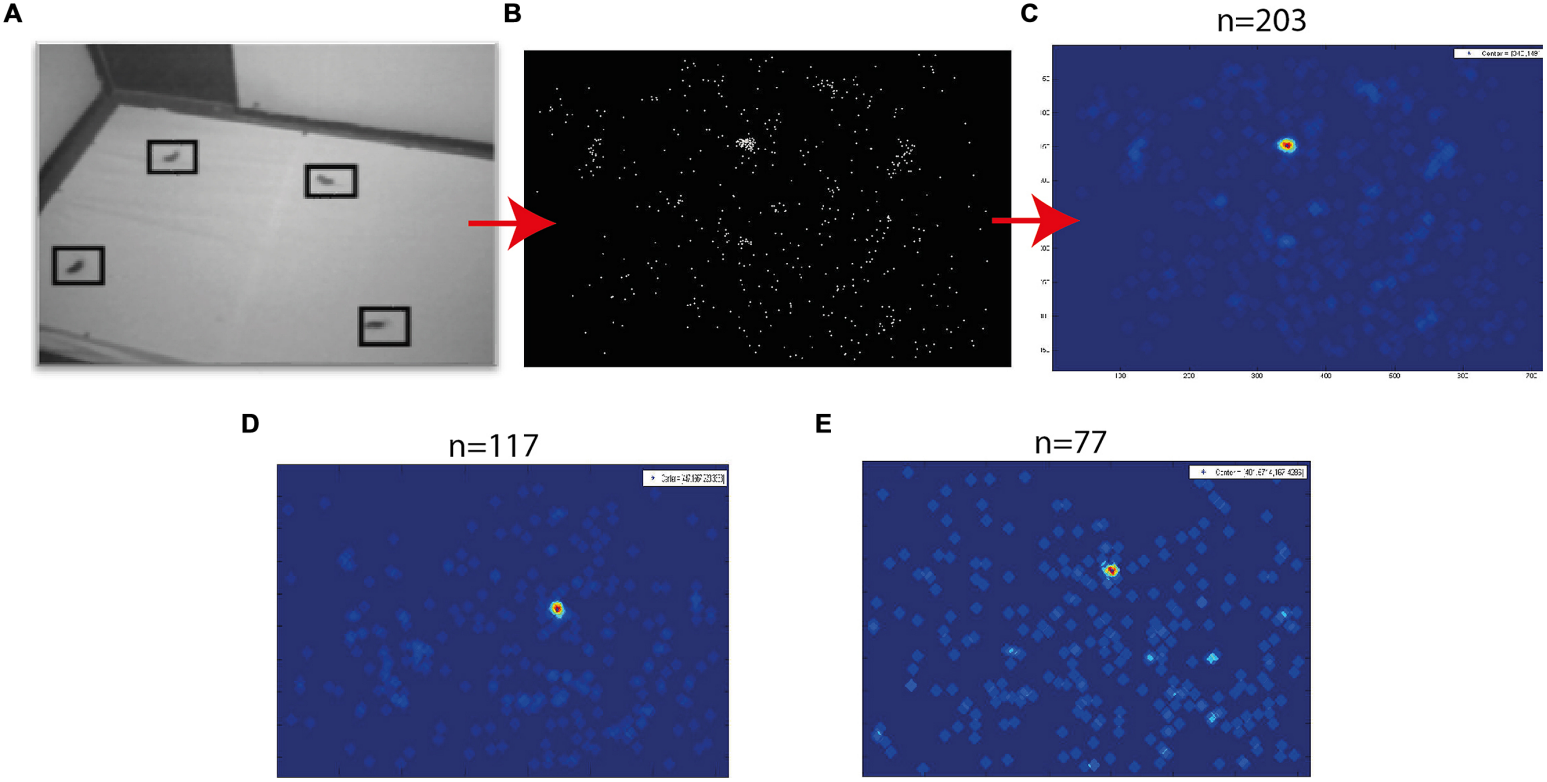
**Target probability maps. (A)** An example of a single fixation frame. Four targets are marked by squares. **(B)** A plot showing all marked targets collected from a single experimental day. **(C)** The density function of the points in **(B)**. Red colors indicate high density and blue low density. **(D,E)** Two additional examples of density functions obtained in different experimental days. The numbers above panels **(C–E)** designate the number of fixation frames analyzed to obtain this image.

**FIGURE 4:**
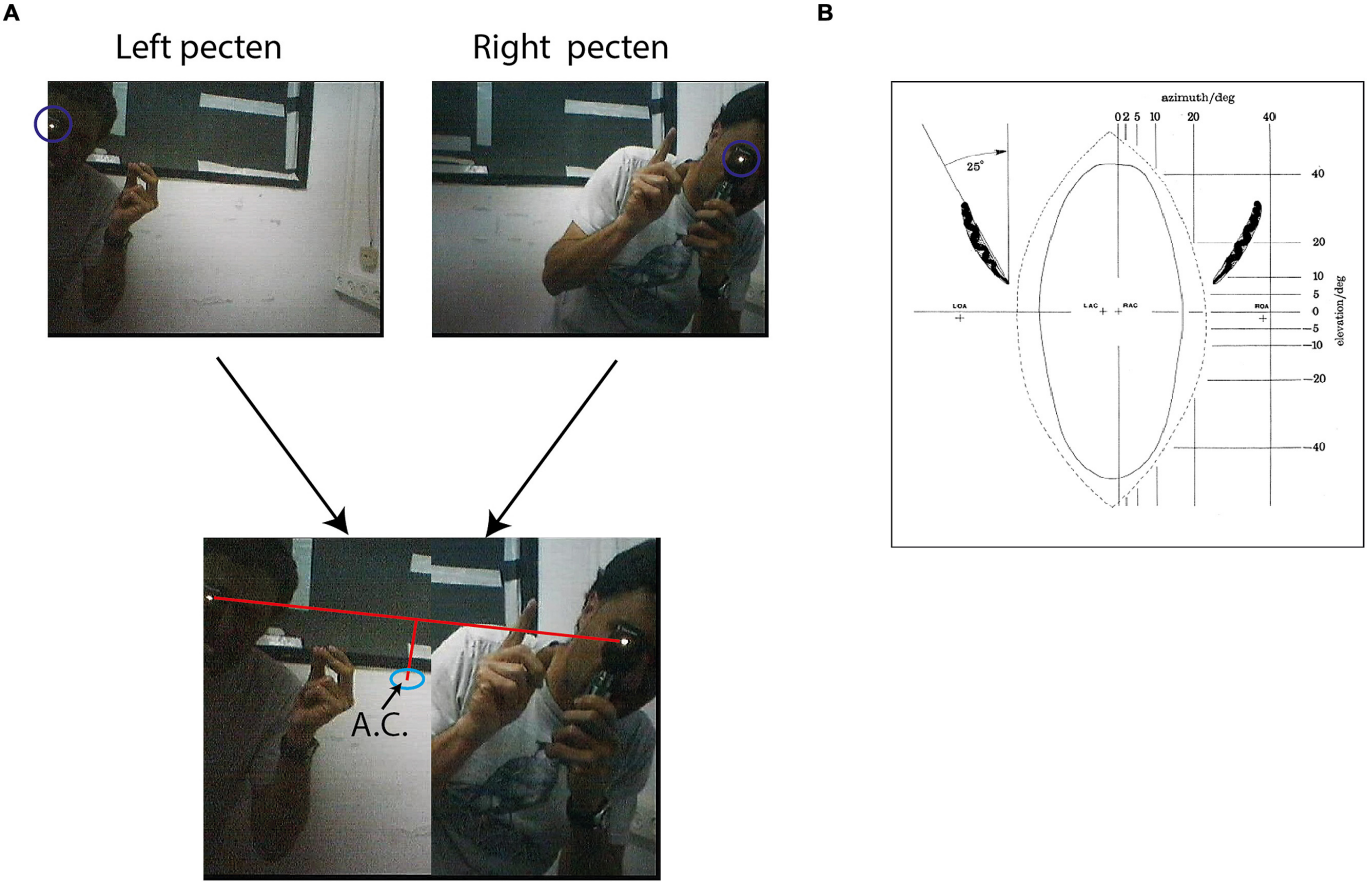
**Mapping of area centralis. (A)** Two frames from the video sequence are shown above. One frame shows an experimenter standing about 1 m from the owl viewing with an ophthalmoscope the superior tip of the left pected and the second from the experimenter viewing the superior tip of the right pecten. The beam of the ophthalmoscope in both cases is marked by a circle. Below the two half frames are combined to one image. A line is drawn between the two beams and the point that is 7° below the mid-point of the line is calculated. This point, marked by a circle, is the estimation of area centralis. **(B)** A scheme showing the projection of the pectens (left and right) on a calibrated screen in front of the owls. The plus marks at the center show the calculated projections of left area centralis (LAC) and right area centralis (RAC). The plus marks at the sides show the projections of the left optical axis (LOA) and the right optical axis (ROA). Positions of LAC, RAC, LOA, and ROA were estimated based on average distances from pectens obtained by analysis of whole-mounted retinas. Figure with permission from Pettigrew (1979).

Fixation frames were analyzed manually. All observable targets and speaker locations were marked with a mouse curser, and the coordinates in the frame were saved (**Figure 3A**). To estimate the coordinates of the functional fovea, all target locations from one experiment were collapsed in one graph (**Figure 3B**), and a density function (the density of the points in each pixel) was calculated to estimate the probability for targets in pixel space (**Figure 3C**). To map the projection of area centralis onto the video frame, a prominent retinal landmark in birds called pecten oculus (Wathey and Pettigrew, 1989) was used. The pecten oculus is a pigmented structure covering the entrance of the optic nerve to the retina. Therefore it is easily viewable with an ophthalmoscope as a dark structure on the highly reflective background of the retina (Netser et al., 2010). We mounted the camera on the owl’s head. Then the owl was held by hand, with its head fixed pointing straight ahead. Another experimenter, who was standing a meter away from the owl, viewed the eye of the barn owl through an ophthalmoscope, and adjusted the position of the ophthalmoscope relative to the owl’s head until viewing the superior tip of the pectin (see **Figure 4B** for an illustration of the pecten). When this was achieved the experimenter marked the video frame by snapping his fingers and moved to the second eye. The relevant video images were extracted offline, and the positions of the ophthalmoscope beam viewing the two pectens were marked manually (see **Figure 4A**) to obtain the coordinates of the projection of the retinal landmarks on the video frame. Since the owls do not move their eyes considerably, the coordinates of the retinal locations are fixed throughout the experiment.

Distances between targets were measured in pixels. Conversion to distance in centimeters was estimated based on the number of pixels recorded in a 10 cm line at the center of the arena and the center of the video frame (30 pixels per 10 cm). Distortion errors due to camera optics and non-equal distances to positions on the arena (keystone distortions) were estimated to be mostly less than 25% and were ignored in this analysis. Since the positions of the targets and speaker were shifted in the arena between different experimental sessions, ignoring these distortions is not expected to create any systemic biases in the results.

Conventional statistical methods were used to assess significance of results. A *p*-value smaller than 5% was considered significant.

## RESULTS

### ANATOMICAL STRUCTURE OF FUNCTIONAL FOVEA

In the first part of this study, we ask whether owls shift their gaze toward targets of interest so as to view the target image on the retinal area centralis. On each experimental day, the OwlCam was first attached and the coordinates for the right and left pectens were mapped as described in the Section “Materials and Methods.” After mapping the pecten tips, the owls were free to stand on the perch. Three to five food items were scattered on the arena, and the owl was left in the room for a period of about half an hour. During this time, the video signal was saved continuously for offline analysis.

A typical video sequence consisted of a series of abrupt head motions (head saccades), each head saccade terminating in a stable period (fixation period). **Figure 2** shows the frame-to-frame correlation function (see Materials and Methods) over a period of 20 s in one such typical experiment. It can be seen that the sequence is composed mostly of stable periods (correlation indices close to 1), but every few seconds, the correlation indices drop abruptly below one, indicating a gaze shift to a new location. For analysis, a single frame was extracted from each stable period (fixation period). Fixation frames in which at least one food item was observed were taken for further analysis while the others were discarded. The positions in the frames of all observed food items were marked manually (**Figure 3A**) and pooled together to create a distribution of food items on the video frame (**Figure 3B**). The probability of each pixel to contain a food item was thus estimated by the density function (**Figure 3C**). It can be seen that the density function resulted in a single most probable point. This pattern was observed consistently (see **Figures 3D,E** for two more examples). In most cases, 10–20 min of recording of spontaneous scan behavior (corresponding to about 80–200 fixation periods) were sufficient to expose such clear, single, most probable point. These results verify the results from previous studies (Ohayon et al., 2008; Harmening et al., 2011) that during a search task, owls scan their environment in a serial manner and repeatedly direct their gaze in a way that brings targets of interest to a specific retinal position, which we refer to as the functional fovea. Since the OwlCam moves with the owl’s head and the eyes are fixed in the head, the functional fovea does not change its position in the video sequences regardless of the owl’s movement.

It is hypothesized that the functional fovea corresponds with the area centralis. To demonstrate that this is indeed the case, we measured both the functional fovea and the area centralis in the same video frame. This dual measurement was performed 15 times in three birds. The functional fovea coordinates on the frame were extracted as the point of maximal probability of an average of at least 60 fixation frames (as demonstrated in **Figure 3**). The coordinates of the area centralis on the video image were mapped based on the histological study of Pettigrew (1979), which analyzed whole-mounted retinas and concluded that the retinal area centralis is located, on average, 25° temporal and 7° above the superior tip of the pecten oculus (**Figure 4B**). We therefore measured the position of the superior tip of the pecten with an ophthalmoscope for both the left and right eyes (see Materials and Methods). A line was drawn between the two positions to designate the horizontal plane. The point 7° perpendicularly below the mid-point was registered as the area centralis (**Figure 4A**). The distances between the left and right pectens were estimated to be between 52 and 55°. These numbers are consistent with the measurements of Pettigrew (1979; 25° per each eye) and indicate that the projections of the area centrales of both eyes tend to converge at a single point directly in front of the owl. Thus, a single functional fovea corresponds with both the left and right area centrales. To assess the reliability of the measurement of the projection of area centralis we repeated in seven cases the measurement twice, once before and once after the release of the owl on the perch for half an hour. In each case we measured the distance in pixels between the two measurements. The average distance was 12.8 pixels and the STD 8 pixels.

**FIGURE 5:**
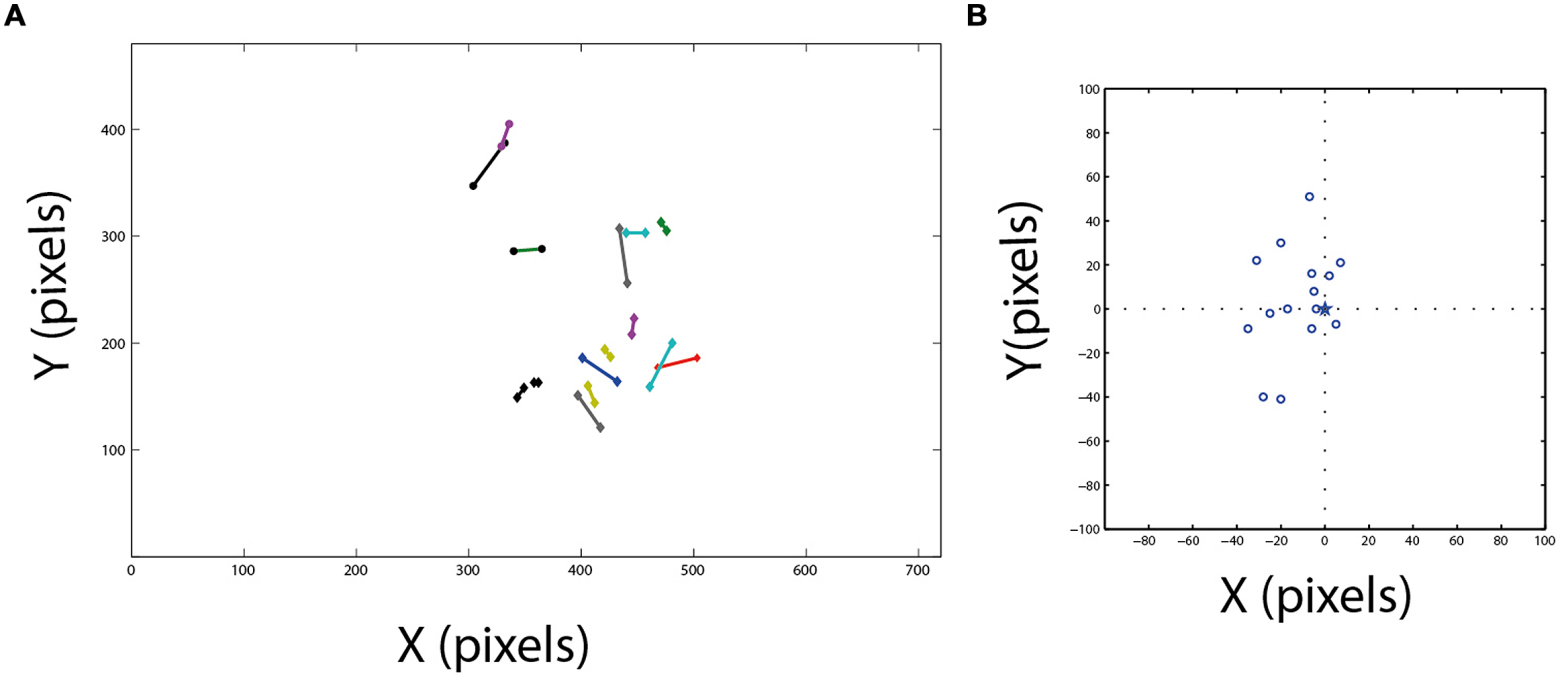
**Comparison between area centralis and the functional fovea. (A)** Fifteen estimated projections of area centralis on the video frame are shown together with the corresponding estimations of the functional fovea. Corresponding pairs are connected with lines. **(B)** The projections of area centralis from panel **(A)** are shown in relation to the position of the corresponding functional fovea. Dash lines mark the zero axes and star marks the position of the functional fovea at the center.

**Figure 5A** shows the location of the functional fovea on the video frame together with the corresponding area centralis. It can be seen that generally there is good agreement between the two independent measurements. The errors are shown in **Figure 5B**, where, for each experiment, the position of the area centralis relative to the position of the functional fovea (0,0 point in the graph) is depicted. The smallest error was 4 pixels and the maximal error was 50 pixels. However, note that the median of the area centralis was 7 pixels biased to the left of the functional fovea (sign test, *n* = 15; *p* < 0.05). This deviation of the area centralis relative to the functional fovea, which corresponds to 2–3°, may arise from small differences in eye positions when the head is immobilized and visible light is shone onto the eye (conditions for measuring the area centralis) compared to active viewing of the environment (conditions for measuring the functional fovea). Interestingly, in the data from Pettigrew (1979), the average mid-position between the two pectens is also shifted to the left of the gaze point by 2–3° [**Figure 4B**, the midpoint between right area centralis (RAC) and left area centralis (LAC)]. Therefore, it seems that when passive and head-fixed, the owls have a tendency to drift eye positions slightly to the left. In the subsequent experiments, where possible, we estimated the point of gaze based on the functional fovea. However, in several cases, not enough spontaneous fixation points were obtained to give a good estimation. In these cases, the area centralis was used to estimate the point of gaze.

### AUDITORY EFFECTS ON VISUAL SEARCH

We used the same owls in the second part of the research. The owls performed an active visual search task with food items as before, however, in this case four food items were scattered in the arena. Each food item was positioned in one quadrant of the arena, and a loudspeaker was hidden under the cloth in one of the quadrants so that the food item in the same quadrant was closer to the loudspeaker but never closer than 10 cm (30 pixels). During each session which was 10–15 min long, a sound stimulus was occasionally emitted from the speaker (see Materials and Methods). About 6–10 sounds were emitted during a single session. After each session, the curtain was drawn, the speaker moved to another quadrant and the positions of the food items were moved slightly for the next session. Each owl performed 2–3 sessions during one experimental day. We analyzed the responses to about 800 sounds in 85 sessions.

**FIGURE 6:**
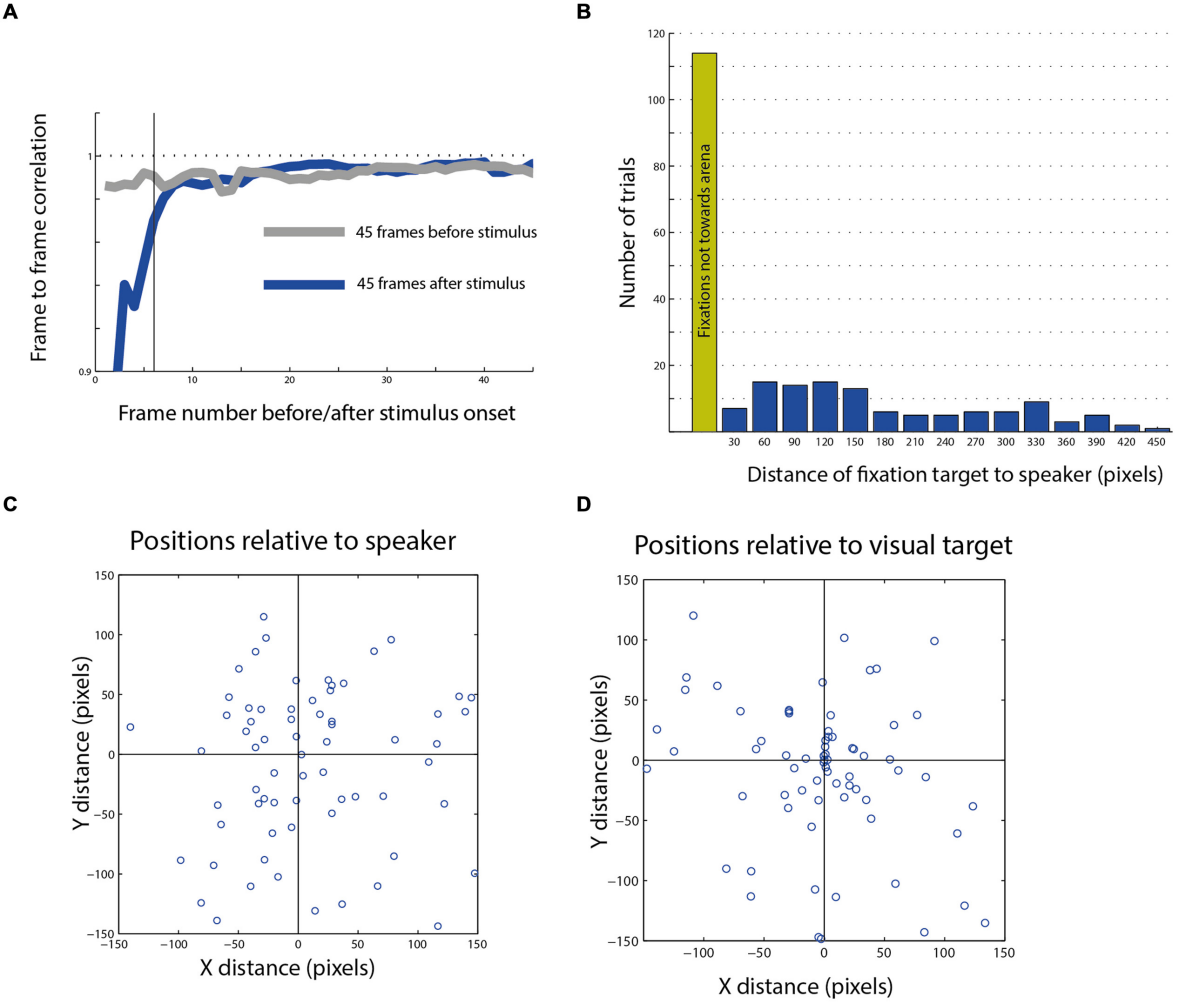
**Post stimulus head saccades end-points. (A)** Average frame to frame correlation graph from 45 frames after the stimulus compared with the average graph from the 45 frames before the stimulus. The dashed horizontal line indicates a correlation of 1. The dark vertical line indicates the sixth frame after stimulus onset, after which the effect of the trigger signal on the correlation function disappears. **(B)** The distribution of the distances of fixation points from speaker location of all movements toward the arena (blue columns). The green column shows the number of fixation points that landed outside of the arena. **(C)** A scatterplot showing the population of fixation end-points which landed in the general area of the speaker plotted with relation to the speaker location (0,0 point). **(D)** The same population as in **(C)**, plotted with relation to the visual target closest to the speaker (0,0 point).

The responses were analyzed offline and first divided into two groups, one in which the fixation point did not change in the 1.5 s after the onset of the sound stimulus (considered as no response and omitted from further analysis) and a second in which a head saccade was elicited in the 1.5 s after the stimulus (considered as a response to the auditory stimulus). Overall in about 35% of stimulus presentations, a head response was induced after stimulation. However, as mentioned earlier, the owls searched the room spontaneously by head saccades even without the sound. Therefore, to examine whether the presence of the sound increased the probability of a head saccade, we averaged the frame to frame correlation function in the 1.5 s following an auditory stimulus and compared it with the average correlation function in the 1.5 s before the auditory stimulus. The average correlation function is an estimation of the saccade probability; the smaller the average correlation, the higher the probability of a saccade. **Figure 6A** shows the average correlation function from 172 stimulus presentations in two owls. A typical delay to an acoustical evoked saccade is 150–300 ms (Whitchurch and Takahashi, 2006), therefore, effects happening in the first six frames unlikely to be attributed to the sound. Examining the probabilities from frame 6 onward, no apparent difference can be seen between the probability of head saccades before and after the stimulus. The lower correlations in the first six frames are attributed to the jitter of the synchronization signal in the video (see Materials and Methods) and do not reflect a head motion. Thus, it seems that the auditory stimulation did not affect the rate of spontaneous head saccades.

Nevertheless, we continued analyzing the targets of the head saccades following the stimuli to explore the possibility that even though the probability of eliciting a saccade is not affected by the auditory stimulus, the choice of the next target is. Among the trials in which a head response was measured in the 1.5 s following the stimulus (*n* = 305), in about half, the gaze changes landed outside the arena (*n* = 161), in many cases attracted toward salient features in the room such as the door knob or the window frame. Only 144 saccades were directed toward the arena. Those latter saccade end points were the subject of subsequent analysis.

The histogram in **Figure 6B** shows the distances between the speaker location and the fixation points on the arena from all three owls. We define a radius of 50 pixels (about 15 cm) from the center of the target as a hit response. This relatively broad circle around the target was chosen to encompass the errors from mapping the area centralis and the errors expected due to different view angles. Only 6% of all responses were considered hits to the speaker. Thus, it seems that the speaker location was a poor attracting point for the final gaze position. This is despite the fact that owls can pinpoint sound sources accurately (Knudsen et al., 1979) and that the sounds were clearly audible and consisted of sound playbacks of objects from the owl’s natural environment. **Figures 6C,D** show the distribution of the gaze end-points of 108 head saccades in which the speaker location was observable at the end-point frame. The same data are plotted in panel C with relation to the location of the speaker and in panel D with relation to the location of the visual target closest to the speaker. It is apparent that the position of the visual target tends to attract gaze while the position of the speaker does not.

**FIGURE 7:**
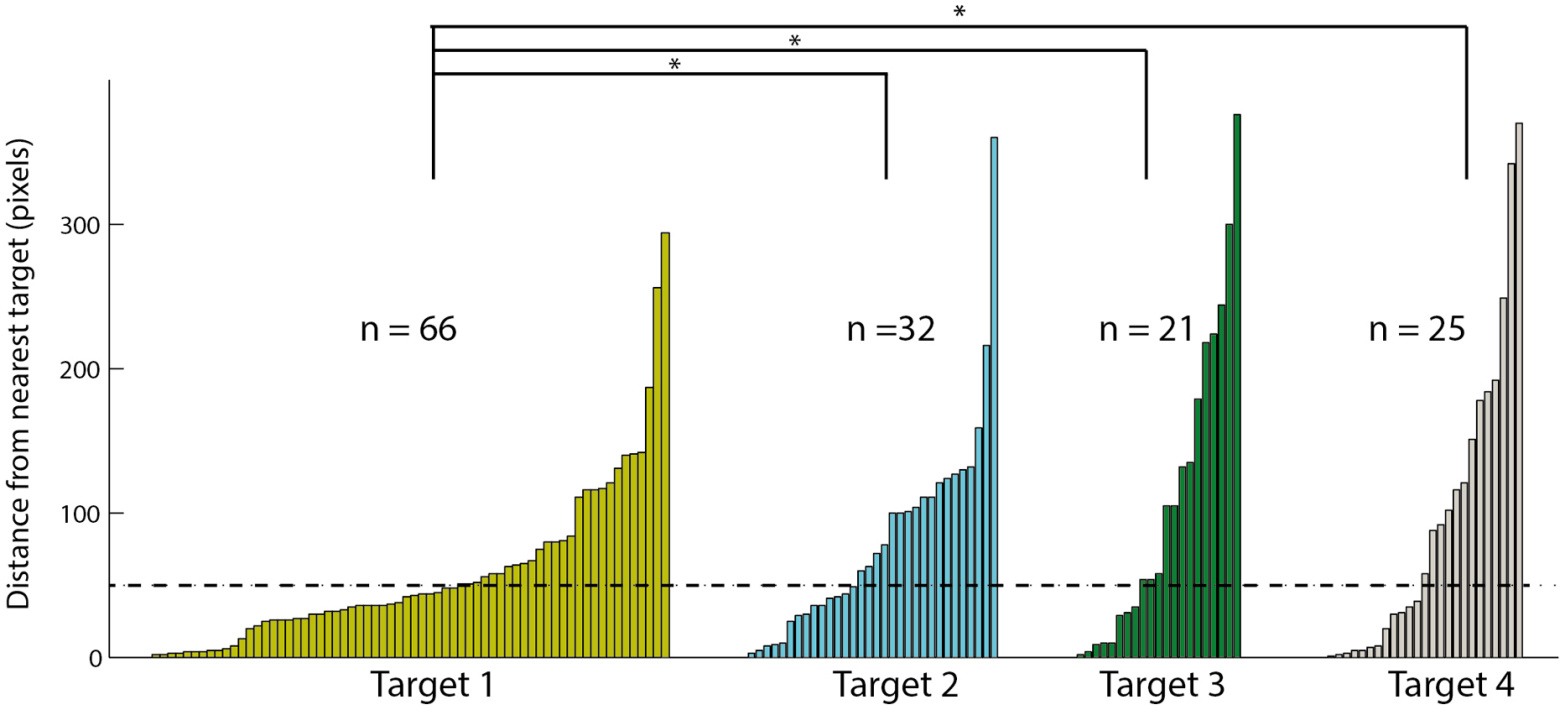
**The distances to the nearest visual target of all fixation points toward the arena.** Data is divided to four targets based on the distance of the target to the speaker, target one is the closest to the speaker and target four the furthest. Within each target the results are sorted according to the distance, smaller distances on the left and larger distances on the right. Asterisks designate a significant (*p* < 0.05) difference in the average distance. The dashed line indicates the 50 pixels criterion line.

To examine the tendency of choosing the different targets, we ranked the targets according to distance from the speaker: the target closest to the speaker was ranked 1 and the target furthest from the speaker was ranked 4. Note that in each session, the speaker location was shifted and therefore the targets ranks were updated accordingly. In the 144 saccades following a stimulus that were directed to the arena, we measured the distance in pixels between the gaze point and the closest target. The data are shown in the histogram in **Figure 7**. Sixty-six of the saccades ended in a position closest to target 1, 32 to target 2, 21 to target 3, and 25 to target 4. This distribution significantly favored the target closest to the speaker location [χ^2^(3) = 35.05; *p* < 0.05]. In addition, the mean distance of the head saccades closest to target 1 (58.2 pixels) was significantly smaller [*t*(142) = -2.75; *p* < 0.05] from the mean distances to targets 2, 3, and 4 (82.4, 110.2 and 97.2 pixels, respectively). Finally, the number of trials considered hits (below 50 pixels) was 39 for target 1 compared to 14 for target 2, 8 for target 3, and 12 for target 4. These correspond to hit rates of 0.59 (39/66) to target 1, 0.44 (14/32) to target 2, 0.38 (8/21) to target 3 and 0.48 (12/25) to target 4, not significantly different from an equal hit rate to all targets [χ^2^(3) = 1.91; *p* > 0.5].

One concern is that the bias we observed toward the target closest to the speaker may arise from prior behavioral biases that the owls might have (for example the owls spontaneously preferring targets near the door). However, this may only pose a concern if the speaker locations were not equally distributed among the four quadrants of the arena. In our experiments out of 85 recording sessions in 21 sessions the speaker was in the upper left quadrant (close to the door), 27 sessions in the lower left, 14 sessions in the upper right, and 23 sessions in the lower right. To address to what extent this can pose a problem we performed a probability simulation by assuming that the owls’ behavior is independent of the speaker location and simply picking for each trial a speaker location based on the above distribution and a fixation location based on hypothetical distributions of the owls’ behavior. First we simulated the very unlikely but the worst case scenario that the owls only look at the lower left quadrant, which is the quadrant in which the speaker happened to be the most. In this case, out of 10,000 simulations we find the mean number of fixations on the quadrant close to the target to be 45 out of 144 trials with only 5 out of 10000 simulations giving a number above 66 (the value that we measured in our experiments). Any other distribution of the owls’ spontaneous movements resulted with a less mean value and smaller percentage above 66. Thus, it is highly unlikely that the preference we see toward target 1 is due to mere probabilities and uneven distributions.

## DISCUSSION

In this study, we investigated visual-auditory integration during spontaneous visual search behaviors in barn owls. The basic assumption in visual search experiments is that by measuring the probability of a target to attract gaze it is possible to estimate the perceived saliency of the target (Treue, 2003). By measuring gaze probabilities of barn owls in various environments Ohayon et al. (2008) have shown that this assumption holds true for barn owls as well.

Most previous behavioral studies of visual-auditory integration in animals used operant conditioning techniques to train the animals to pinpoint the location of a single modality stimulus and then measure the reaction time and accuracy of responses to unimodal and bimodal stimuli (Stein et al., 1988; Whitchurch and Takahashi, 2006; Schiller et al., 2012). In barn owls, it was shown that the reaction time and accuracy of responses to bimodal stimuli were not better than those of responses to unimodal stimuli. Yet, the response as a whole could still benefit from a bimodal stimulus by enjoying both the faster reaction time of an auditory response as well as the better accuracy of a visual response (Whitchurch and Takahashi, 2006). Thus, this previous study does not provide evidence that owls can improve accuracy or speed by integrating visual and auditory information. It is, however, possible that visual and auditory information is integrated, not for localizing single targets, but rather for enhanced saliency mapping (Talsma et al., 2010). To investigate this possibility, we adopted a different approach: we took advantage of the owl’s natural tendency to scan the environment by abrupt shifts of gaze every few seconds (Ohayon et al., 2008). We first show that by doing so, the owls tend to bring targets of interest (i.e., high saliency) to project onto the retinal area centralis. Thus, by tracking the projection of the area centralis onto the scene, it is possible to estimate the perceived saliency of targets. We then estimated the saliency of targets as a function of their proximity to a hidden loudspeaker that occasionally produced a sound stimulus. Three possible outcomes are envisioned: one, that the owls will respond by turning their gaze to localize the sound-source; two, that the owls will ignore the sounds and continue scanning the environment independent of the sound source; and three, that the owls will not localize the sound source but rather adjust their visual search behavior according to the location of the sound source. The first two possibilities would indicate not a visual-auditory integration but rather a dominance of one modality over the other or competition between the two modalities. The latter possibility would indicate that information from the sound stimulus is integrated with visual information to create a joint functional saliency map (Onat et al., 2007). We have found that the owls tend to look more at the vicinity of the target close to the speaker. This observation is consistent with the third possibility above, but it can also be explained by the first possibility, i.e., that the owls from time to time localized the sound source independent of the visual target. However, if this was the case we would have expected that the mean distance of the head saccades closest to target 1 would be larger compared to the same values for targets 2–4 and that the hit rates on target will be smaller for target 1. In fact, the mean distance to the visual target was smaller for target 1 and the hit rates were not significantly different between the targets. Thus, our results support the third possibility: the visual target closest to the sound source attracted more attention compared to the other targets. These results therefore are consistent with the notion that visual-auditory integration is used for saliency mapping in barn owls.

A similar experimental approach was taken in a study of human visual search (Onat et al., 2007). Human subjects were exposed to images of natural scenes, and the probability of elements in the image to attract eye fixations were measured under three conditions: when no sounds were generated; when a speaker on the right side of the image was playing sounds; and when a speaker on the left side of the image was playing sounds. Using this apparatus, the authors showed that similar to results in this study the saliency of elements in the image are modulated according to their distance from the sound source. Fixations on visual elements on the side close to the sound source were more probable. A common notion in saliency mapping is that saliency is first mapped for individual features such as shape, color, modality, etc., and then the individual computations are combined into a global saliency map that integrates different modalities and features (Itti and Koch, 2000; Treue, 2003). According to this notion, saliency is biased toward the stimulus closest to the loudspeaker since in the global saliency map information is integrated with spatial information from the auditory sense. The similarity between the results of this study and the study of Onat et al. (2007) suggest that similar multisensory strategies are used by humans and barn owls to determine the next target for overt attention. This study, therefore, is in line with several recent behavioral studies in barn owls that point to similarities between humans and barn owls in visual search and attention allocation behaviors (Johnen et al., 2001; Ohayon et al., 2008; Harmening et al., 2011).

One clear observation in our results was that the sound stimulus itself seemed to be of low saliency relative to the visual targets. In most cases, it did not elicit a gaze shift, and when it did, it was seldom toward its source. In fact, it seemed that the probability of changing a gaze shift after the sound was not elevated compared to spontaneous responses (**Figure 6A**). This is somewhat surprising, particularly since barn owls are well known for their accurate sound localization behaviors (Knudsen et al., 1979; Konishi, 2000), in some cases using hearing alone to capture mice in complete darkness (Payne, 1971). This lack of interest in the sound was despite the fact that the sounds were clearly audible, were composed of natural elements, and were very restricted in time. One likely explanation for lack of interest may be that the owls were used repeatedly in the same task. Barn owls habituate dramatically to repeating sounds (Netser et al., 2011). Therefore their responses to the auditory stimulus in this experiment are expected to habituate over time. It is plausible that in natural unfamiliar cases where sounds may carry behaviorally relevant information, the owls will respond considerably more to sudden sounds. In the conditions of the current experiments, the owls learned that the sounds carry no behavioral meaning and were of low saliency. However, although this low saliency was not enough to compete with the saliency of the visual targets, it was still enough to bias their perceived saliency.

In the cases where, following the sounds, the owls changed their gaze toward the general direction of the speaker, the gaze shifts tended to acquire the visual target and not localize the sound source (**Figures 6C,D**). The tendency to shift gaze toward close visual targets and not directly to the auditory source is consistent with the notion of visual capture or ventriloquism (Recanzone, 2009). It is well documented that humans and primates, when confronted with visual and auditory stimuli that are incongruent in space, tend to localize the sound as if coming from the location of the visual stimulus (Recanzone, 1998; Woods and Recanzone, 2004), and accordingly increase the saliency of the visual stimulus location (Spence and Driver, 2000). Visual capture makes sense because in most conditions the reliability of visual localization is larger than the reliability of auditory localization. Although no direct evidence for visual capture in barn owls has been reported, experiments with prismatic spectacles suggest that it does take place (Knudsen and Knudsen, 1989; Gutfreund and Knudsen, 2004). We therefore hypothesize that in clutter environments where visual features are abundant, the owls will show a tendency to acquire visual targets rather than the location of the auditory stimulus. It would be interesting to find out whether the owls will continue to be attracted to the salient visual targets close to the sound source more than to the sound source itself, if the experimental conditions could be varied to make the sound more salient to the owl, for example, by associating the sound with a food reward or by creating more natural unpredicted conditions.

An open question is where in the brain the interactions between visual and auditory signals for saliency mapping take place? In mammals, focus has been drawn mostly to the lateral intraparietal cortex [LIP; Bisley and Goldberg (2003)] and to the SC (Stanford et al., 2005; Fecteau and Munoz, 2006). In birds, the analog forebrain area to LIP is unknown, however, the homolog region to the SC is the OT (Jarvis et al., 2005). The OT in barn owls has been studied extensively, and a series of studies recently point to the notion that activity in the OT reflects the saliency mapping necessary for overt selective attention (Winkowski and Knudsen, 2007; Reches and Gutfreund, 2008; Zahar et al., 2009; Mysore et al., 2010, 2011; Netser et al., 2011). Thus, it is likely that the behavioral results observed in this study reflect activity of tectal neurons. In barn owls, tectal neurons that integrate visual and auditory signals (multisensory neurons) are highly abundant (Knudsen, 1982; Zahar et al., 2009), more common compared to the OT of other avian species (Wang, 2003) or to the SC of mammals (King and Palmer, 1985; Populin and Yin, 2002). It was shown that multisensory neurons in the barn owl’s OT tend to combine visual and auditory signals in a supra-linear manner, if the stimuli are congruent in space and time and if the stimuli are surprising (Zahar et al., 2009). These findings resemble rules of visual-auditory integration found in the SC (Meredith and Stein, 1983). However, the behavioral paradigm of the current study requires a different type of interaction. Here, the auditory stimuli were abrupt and scarce in time, while the visual scene was fixed (in room coordinates). Thus, if the saliency of the scene is expressed in the activity of neurons in the tectal map, we expect the auditory stimulus to modulate the activity in the map over a time span larger than the duration of the stimulus itself. Short-term auditory memory in tectal neurons has been reported for periods of up to a minute and possibly more (Reches and Gutfreund, 2008; Netser et al., 2011). Thus, the substrate for modulating visual activity by short localized auditory stimuli exists in the OT.

## CONCLUSION

In this study we report that auditory information biases spontaneous visual search in barn owls. This suggests that auditory-visual integration takes place at early pre-attentive stages in order to guide spatial attention. These findings are consistent with a model proposed by Itti and Koch (2000) to explain bottom–up mechanisms of visual search in primates. Our findings therefore suggests that similar, pre-attentive, visual-auditory integration takes place in non-mammalian species as well, pointing to the generality of such integration. In nature the visual and the auditory scenes are highly dependent. Thus, it makes sense to integrate the two modalities at early stages to enhance the important behavior of identifying the most salient target for attentional responses.

## Conflict of Interest Statement

The authors declare that the research was conducted in the absence of any commercial or financial relationships that could be construed as a potential conflict of interest.
